# HETEROGENEITY OF PREFRONTAL CORTICAL ACTIVITY DURING WALKING AMONG OLDER ADULTS

**DOI:** 10.1093/geroni/igad104.0551

**Published:** 2023-12-21

**Authors:** Emma Baillargeon, Theodore Huppert, Caterina Rosano, Andrea Rosso

**Affiliations:** University of Pittsburgh, Pittsburgh, Pennsylvania, United States; University of Pittsburgh, Pittsburgh, Pennsylvania, United States; University of Pittsburgh, Pittsburgh, Pennsylvania, United States; University of Pittsburgh, Pittsburgh, Pennsylvania, United States

## Abstract

Higher prefrontal cortical (PFC) activity during simple walking may indicate neural compensation for impaired gait automaticity. On average, older adults have higher PFC activity during walking than younger adults, but the heterogeneity amongst older adults is understudied. We examined PFC activity during simple and dual-task walking in adults aged 65+ (n=173, mean age=72, 62% female). Participants walked quietly and while reciting every-other-letter of the alphabet (dual-task; 4 repsx15 meters). Change in PFC activity from standing was estimated using functional near-infrared spectroscopy. We grouped participants with significantly increased, significantly decreased, or no change in PFC activity from standing to simple walking (t-test, alpha=0.05). We tested for group differences in PFC activity change from simple to dual-task walking, task performance (gait speed, rate of correct letters), age, and cognitive function (trail-making A/B). PFC activity increased from standing to walking in 52 (30%), decreased in 21 (12%), and did not change in 100 (58%) participants. Those who reduced PFC activity from standing to simple walking (vs no change or increased, respectively) walked faster (mean+/-SD=1.12+/-0.17 vs 1.00+/-0.17 0.99+/-0.18m/s; both p=0.014) and had a greater increase in PFC activity from simple to dual-task walking (t-stat=1.96+/-2.2 vs 0.49+/-1.53 vs -0.26+/-1.43; p=0.008, p=0.0006). Those who increased PFC activity from standing to simple walking had a smaller PFC change (vs no change) from simple to dual-task walking (p=0.005). Age, alphabet performance, and cognitive function did not differ across groups. Differences in PFC activity during simple walking may contribute to differences in task performance and capacity for walking challenges.

